# Features of reactive cysteines discovered through computation: from kinase inhibition to enrichment around protein degrons

**DOI:** 10.1038/s41598-017-15997-z

**Published:** 2017-11-27

**Authors:** Nicholas J. Fowler, Christopher F. Blanford, Sam P. de Visser, Jim Warwicker

**Affiliations:** 1 The Manchester Institute of Biotechnology, The University of Manchester, 131 Princess Street, Manchester, M1 7DN United Kingdom; 2 School of Chemistry, The University of Manchester, 131 Princess Street, Manchester, M1 7DN United Kingdom; 3 School of Materials, The University of Manchester, 131 Princess Street, Manchester, M1 7DN United Kingdom; 4 School of Chemical Engineering and Analytical Science, The University of Manchester, 131 Princess Street, Manchester, M1 7DN United Kingdom

## Abstract

Large-scale characterisation of cysteine modification is enabling study of the physicochemical determinants of reactivity. We find that location of cysteine at the amino terminus of an α-helix, associated with activity in thioredoxins, is under-represented in human protein structures, perhaps indicative of selection against background reactivity. An amino-terminal helix location underpins the covalent linkage for one class of kinase inhibitors. Cysteine targets for S-palmitoylation, S-glutathionylation, and S-nitrosylation show little correlation with pKa values predicted from structures, although flanking sequences of S-palmitoylated sites are enriched in positively-charged amino acids, which could facilitate palmitoyl group transfer to substrate cysteine. A surprisingly large fraction of modified sites, across the three modifications, would be buried in native protein structure. Furthermore, modified cysteines are (on average) closer to lysine ubiquitinations than are unmodified cysteines, indicating that cysteine redox biology could be associated with protein degradation and degron recognition.

## Introduction

Cysteine reactivity has long been recognised as a key factor in the activity of many proteins, including thiol disulphide oxidases/isomerases. An example is the thioredoxin family^1^, in which cysteine reactivity determines biological function across a wide range of redox potentials, based on amino acid variation around common location at the amino-terminus of an α-helix^2^. A correlation between redox potential and cysteine pKa has been established^3^, and predictive models based on pKa calculations have been used to model variation within the family^4–6^.

From high-throughput proteomics, it has become evident that cysteine reactivity is generally important in proteins, with a variety of cysteine sidechain modifications^7^. Influences on amino acid susceptibility to post-translational modification range from intrinsic reactivity of a particular amino acid sidechain (largely the case for many members of the thioredoxin family) to detailed amino acid sequence specificity (for example in human protein kinases). For a modification mediated by enzyme catalysis, reliance on the intrinsic reactivity of a sidechain is often reduced and sequence recognition plays a major role. With cysteine modifications, as mass spectrometry and detailed biochemical studies^8^ reveal their presence, issues around how these modifications are encoded and carried out are largely unresolved. High-throughput proteomics datasets are being used to identify post-translationally modified cysteines^9^, including the addition of palmitate, glutathione, or an NO group. Underlying factors for these modifications are then sought, leading to the development of bioinformatics prediction tools with respect, for example, to palmitoylation^10^, glutathionylation^11^, and nitrosylation^12^. Prediction tools rely mostly on populations of sequence motifs around modified sites^13^, whilst the question of biophysical influence on modification, analogous to modulation by charge interactions in the thioredoxin family, remains open. A recent study of three types of cysteine modification, followed by sequence and structural analysis of the modified sites, reports that biophysics appears not to play a significant role^9^.

Three of the most numerous modifications in mass spectrometric data, presumably reflecting important roles in nature^14^, are S-glutathionylation, S-palmitoylation, and S-nitrosylation. Functionally, reversible S-glutathionylation may be used to protect reactive cysteines, under oxidative stress^15^. S-palmitoylation is an example of fatty-acylation of proteins, though to be functional in targeting to a membrane^16^, mediated by a family of palmitoyl transferases (PATs), containing DHHC domains that are named after a conserved amino acid motif. Protein S-nitrosylation has a variety of emerging roles in signalling and disease^17^, and proposed mechanisms of modification include the use of direct NO or nitrosylating equivalents and trans-nitrosylation^18^.

Reactive cysteines are an emerging pharmaceutical target, in particular those close to active sites, exemplified by the use of irreversible inhibition for the T790M mutant of human epidermal growth factor receptor (EGFR)^19^. A prime example is covalent modification of C797^20^. Susceptibility to modification is presumably mediated by the cysteine sidechain accessibility and reactivity as well as the complementarity of the surrounding active site to the associated drug-like moiety^20^.

Methodologies for pKa and reactivity prediction are here applied to the high-throughput proteomics data that are accruing for cysteine modifications. First, a representative set of human proteins from the structural database are examined for cysteine location, finding that they are under-represented at helix amino-termini, consistent with selection against reactive cysteines in general. Next, in a set of human kinase structures, cysteines at helix amino termini are consistently predicted as reactive, including C797 of EGFR. Looking more generally at cysteine post-translational modifications (PTMs, palmitoylation, glutathionylation, nitrosylation), a strong predicted preference for reactive thiolate is not evident, but a third to a half of the sites that can be structurally annotated have zero solvent accessibility. Expanding to study sequence, net charge is enriched in a sequence window around modified sites, to an extent that depends on modification type. These results have implications for both the mechanisms of cysteine modification (and whether the thiolate form is preferred), and the folding status of protein targets. The latter aspect is highlighted by further analysis showing an enrichment for cysteine modification sites lying close to sites of lysine ubiquitination.

## Results

### Cysteine in human proteins is under-represented at the amino termini of α-helices

**Figure 1:**
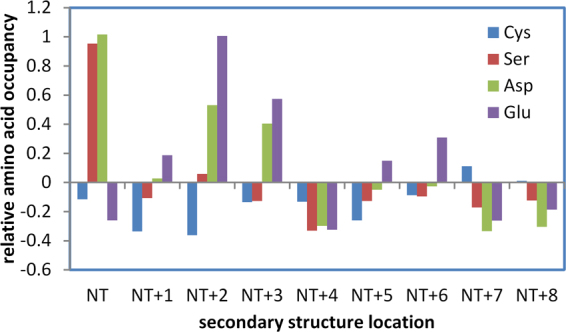
Cysteine is depleted at α-helical amino-termini in human proteins. Enrichment (positive) or depletion (negative) of Cys, Ser, Asp, Glu, compared to overall propensity of each amino acid in the hu1627 dataset, is shown at locations close to amino-termini (NT) of α-helices.

The hybrid Finite Difference Poisson-Boltzmann (FDPB) and Debye-Hückel (DH) method (termed FDDH) is effective for pKa prediction of cysteines in the thioredoxin family, capturing a range of pKa deviation that relates directly to biological function^5^. An important feature in this family is location of the reactive cysteine at the first position within an α-helix, favouring the thiolate form of the cysteine sidechain. If such an arrangement generally increases reactivity of cysteine, then avoidance of non-specific reactivity could lead to selection against it across the proteome. Figure 1 shows the relative propensities of Cys, Asp, Glu and Ser at locations adjacent to the amino-termini of α-helices (independent of any other measures, such as accessibility), within the hu1627 set of human protein structures. Asp is well-known as a helical N-cap residue^21^, borne out here, with Ser showing a similar enrichment, presumably due to hydrogen bonding potential with helix termini NH groups. Glutamic acid is enriched towards the helix amino terminus but not at the first helical residue. It is assumed that Glu sidechain geometry for interaction with helix NH groups is less optimal than for Asp at the first residue. Interestingly, Cys is depleted rather than enriched at the first helical location. This could arise from polar thiol interactions that are of lower magnitude than those formed by the Ser hydroxyl, but it is also consistent with avoidance of an environment that will lower its pKa and (non-specifically) increase cysteine reactivity to modification.

### Helix amino terminal cysteines are predicted to be more reactive

**Figure 2:**
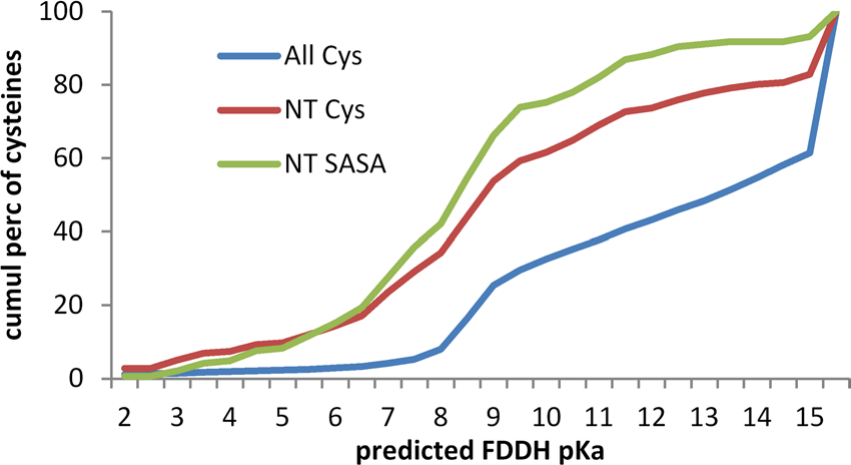
Predicted pKas of cysteines in the hu1627 dataset. Three subsets of cysteines are shown in plots of cumulative percentage distributions: all cysteines in these structures (All Cys, 8559), only Cys located at the first position in an α-helix (NT Cys, 216), and the NT subset further restricted to include only those with SASA > 0.1 Å^2^ (NT SASA Cys, 145). Each set contains cysteines that have predicted pKas > 15. These tend to arise for buried groups with high desolvation penalties, and are accordingly less evident for the NT SASA Cys set.

Given the relative reactivity of active site cysteines in the thioredoxin family, it might be expected that Cys in the hu1627 set (defined in the Methods section) that are at the amino termini of α-helices would be enriched for lower predicted pKas. This is indeed the case (Fig. 2), with the distribution of predicted pKas for Cys at the first α-helical position (216 instances) down-shifted from that for all Cys (8559). Since there is little difference for the distributions of Cys at the first α-helical position and those at this location and also with solvent accessible surface area (SASA) > 0.1 Å^2^ (145), the overall down-shift is not due to sidechain burial from solvent. These results suggest that, in terms of nucleophilicity, Cys residues at the first position in an α-helix are relatively reactive.

### Reactivity of cysteines at α-helix amino termini in human kinase drug targets

**Figure 3:**
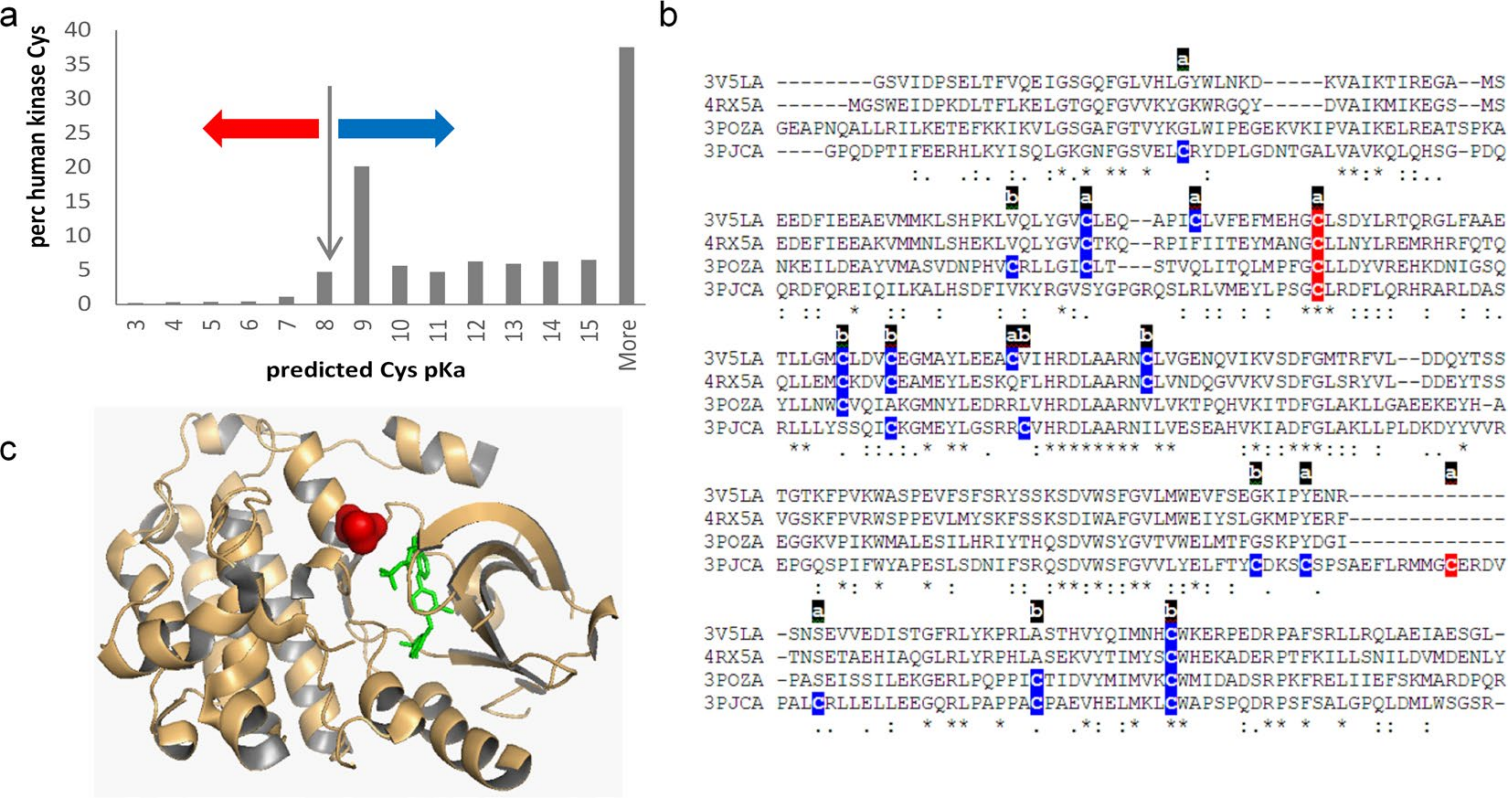
Cysteine modification in human kinases. (**a**) Predicted cysteine pKas for a non-redundant set of human protein kinases, with red and blue arrows marking pKa decreased or increased, respectively, from the model compound pKa of cysteine (8.3). (**b**) Kinase domains of 4 kinases (3v5l A, 4rx5 A, 3pozA, 3pjcA) are aligned, with colour coding of cysteines according to a predicted lowering (red, more reactive) or raising (blue) of the sidechain pKa. Accessibility of each cysteine is marked as ‘a’ (accessible) or ‘b’ (buried). (**c**) EGFR kinase domain is shown for 3poz chain A, with inhibitor (green), and reactive cysteine site (red) at an α-helix amino-terminus.

Cysteine pKas were predicted with the FDDH method for a set of 3D structures that map to a unique set of sequences for human protein kinases. Additionally, SASA values and secondary structure locations were recorded for these single chain calculations. The distribution of predicted pKas peaks around the unperturbed Cys pKa (8.3), and at the high values that are indicative of buried groups with large dehydration penalties (Fig. 3a). Cysteine sidechains for which negative charge is stabilised or destabilised relative to the unperturbed pKa are indicated. The effect of an amino-terminal α-helix capping location is shown by the percentage of these sites predicted to have the thiolate form stabilised (pKa < 8.3), at 58.8% (of 68), compared with the equivalent 10.6% for all 2814 cysteines in the dataset.

For a site of established pharmacological relevance, C797 in the human EGFR kinase, (3poz^22^,) and the equivalent site in three other tyrosine receptor kinases (3pjc^23^, 3v5l^24^, 4rx5^25^), a predicted reactivity colour scheme is marked on the kinase domain sequences in Fig. 3b. The accessibility of each cysteine site is also marked, based on a SASA threshold of 1.0 Å^2^. Of the 5 cysteines marked as relatively reactive according to predicted pKas, 4 are at the site equivalent to C797 of EGFR kinase, and the singleton is C1040 in 3pjc (chain A), with a predicted pKa of 8.2, just under the 8.3 unperturbed value. For 3poz, the reactive cysteine is displayed in the structure, with an inhibitor marking the active site (Fig. 3c).

Cysteine 797, a target for irreversible inhibition of kinase activity, is located at the amino-terminal capping position of an α-helix, in common with reactive cysteines of the thioredoxin family. Whilst the use of an adventitious reactive sidechain is only a part of the requirement for inhibitors targeted at cysteine, alongside pocket complementarity for a drug-like moiety, prediction of cysteine reactivity could be a useful tool in drug design for protein kinases and other targets. In order to assess the consistency of pKa predictions across EGFR kinases, the condition for single representatives at 100% sequence identity was withdrawn and pKas predicted with the FDDH method for the C797 site in 115 structures (with ligands excluded). Average predicted pKa is 7.7, with a standard deviation of just 0.5, i.e. this site is consistently predicted as reactive.

### Cysteine PTM sites in protein structures are only slightly enriched for positively-charged environment, but a surprisingly large proportion are buried from solvent

**Figure 4:**
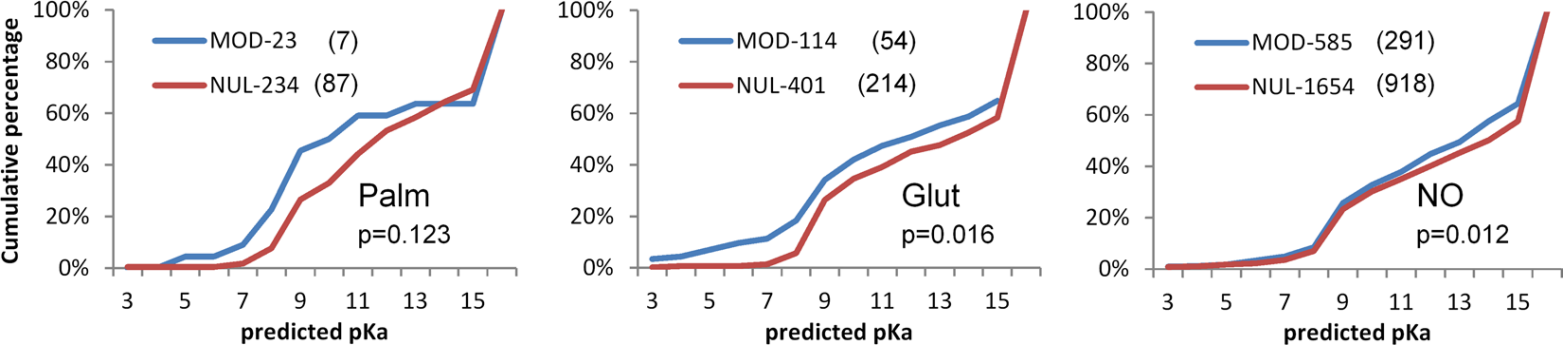
Predicted pKas and solvent accessibility for structurally annotated cysteine PTM sites. For each of the S-palmitoylated, S-glutathionylated and S-nitrosylated structural sets, FDDH predicted pKas are shown for modified (MOD) cysteines (blue) and unmodified (NUL) cysteines in the same proteins (red). Mann-Whitney test two-tailed p-values for separation of modified and unmodified set pKas are given, with numbers in the sets appended to the MOD, NUL descriptors, followed in parentheses by the number of cysteines in each set for which SG atoms are buried (zero SASA). High desolvation penalties associated with burial lead to pKas > 15 for some cysteines.

We next tested whether the charge environments of cysteines are reflected in propensity for post-translational modification. Predictions of cysteine pKas are shown in Fig. 4 for positive and (presumed) negative sites of modification, for S-glutathionylation, S-nitrosylation and S-palmitoylation. Numbers of sites that can be structurally annotated are much smaller than the total available from mass spectrometry, and trends are not clear-cut. For S-palmitoylation there appears to be some enrichment for lower predicted pKas at modified sites, but the numbers are low and the result is not statistically significant. With larger numbers, the small shift to lower pKa is significant for S-glutathionylation, whilst for S-nitrosylation, a small difference is apparent and statistically significant, but is at higher pKa values rather than the more reactive range. Cysteines at amino termini of α-helices are not prominent in the modified sets, 0 of 23 for S-palmitoylation, 9 of 114 for S-glutathioylation, and 18 of 585 for S-nitrosylation.

The most striking aspect of these calculations is the extent to which modified sites are buried from solvent (Fig. 4). In order to exclude sites that are at the surface with only a small part of their surface solvent accessible, the SASA threshold was taken as zero. Even so, the fraction of cysteines that are buried is almost as high for the modified sites as the unmodified sites (S-palmitoylation 0.30 fraction modified, 0.37 unmodified; S-glutathionylation 0.47, 0.53; S-nitrosylation 0.50, 0.56). A small difference between the fraction of modified and unmodified sites that are buried may underlie the shift in predicted pKas at higher values, since buried cysteines (of which there are slightly more in the unmodified set) will tend towards higher pKas. High predicted pKas are notional, since a protein will not experience these pH values, and are used here simply as an indicator of physicochemical environment. It is possible that some proteins may be able to fold around an incorporated NO group, but less likely for glutathione, and implausible for the palmitoyl group.

### Cysteine PTM sites are not predicted to be enriched in intrinsically disordered regions, and are enriched in abundant proteins

**Figure 5:**
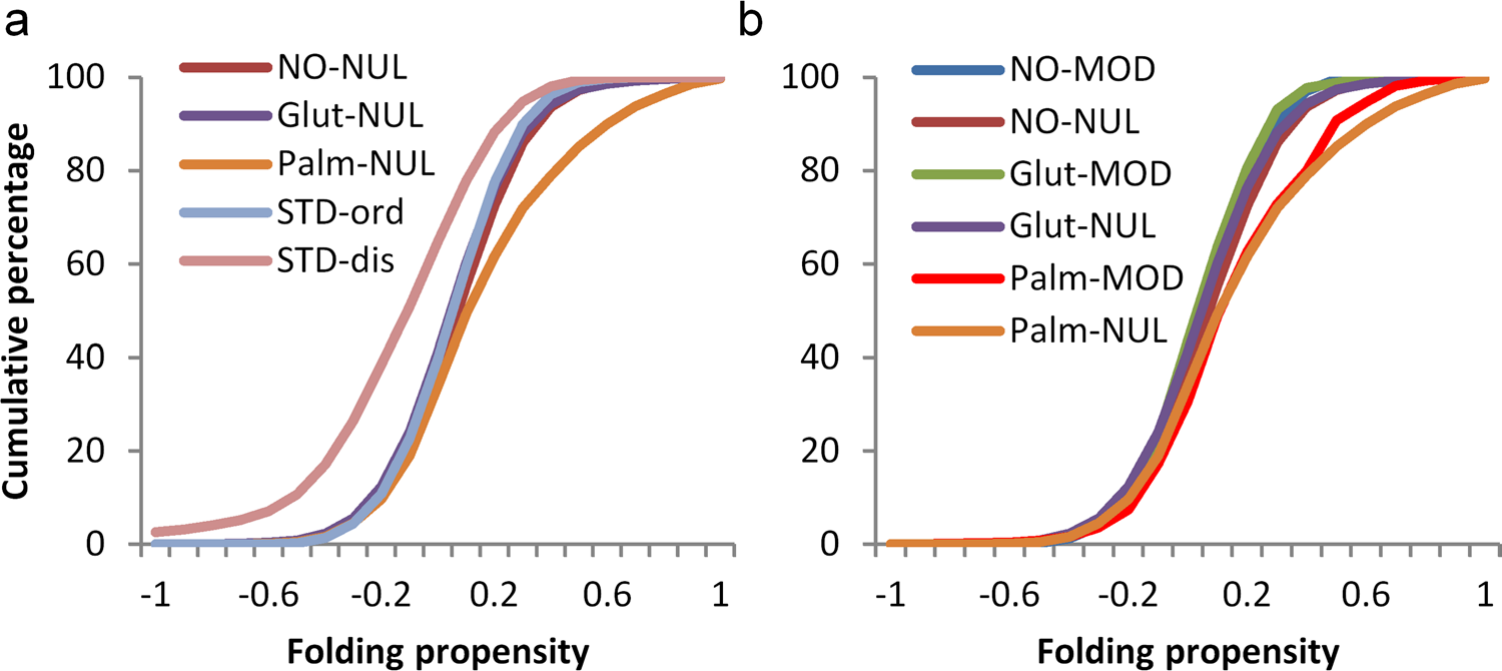
Sites of cysteine modification are not in regions predicted to be disordered. (**a**) Predicted folding propensity (in 21 amino acid windows) is shown for benchmark standard sets (STD) of ordered and disordered proteins. Unmodified cysteine sites in sets of proteins that are modified either coincide with the ordered benchmark set or (S-palmitoylated proteins) include regions with greater folding propensity. (**b**) Cysteine PTM sites approximately coincide with their cognate unmodified sites, with no clear trend towards predicted disorder.

The most evident general biophysical property of phosphorylation sites (largely on serine, threonine, and tyrosine sidechains) is that they are enriched in regions of protein disorder, and this observation forms the basis for a prediction scheme^26^. To test whether the same is true for modifications of cysteine, fold propensity^27^, averaged over 21 amino acid windows, was calculated for benchmark sets of ordered and disordered proteins^28^. The resulting distributions were compared with those obtained for unmodified cysteine sites in the three sets of proteins that contain modified sites (Fig. 5a). S-glutathionylation and S-nitrosylation sites are close to the ordered benchmark, with S-palmitoylation shifted towards a higher predicted folding propensity. The disordered benchmark set is left-shifted compared with all other sets, as expected. Figure 5b shows that cysteine modification sites are close in fold propensity to their respective unmodified sites, and thus cysteine modifications are predicted to lie, generally, in structured protein regions. Accentuated folding propensity for S-palmitoylation sites presumably relates to their classification as peripheral membrane proteins.

**Figure 6:**
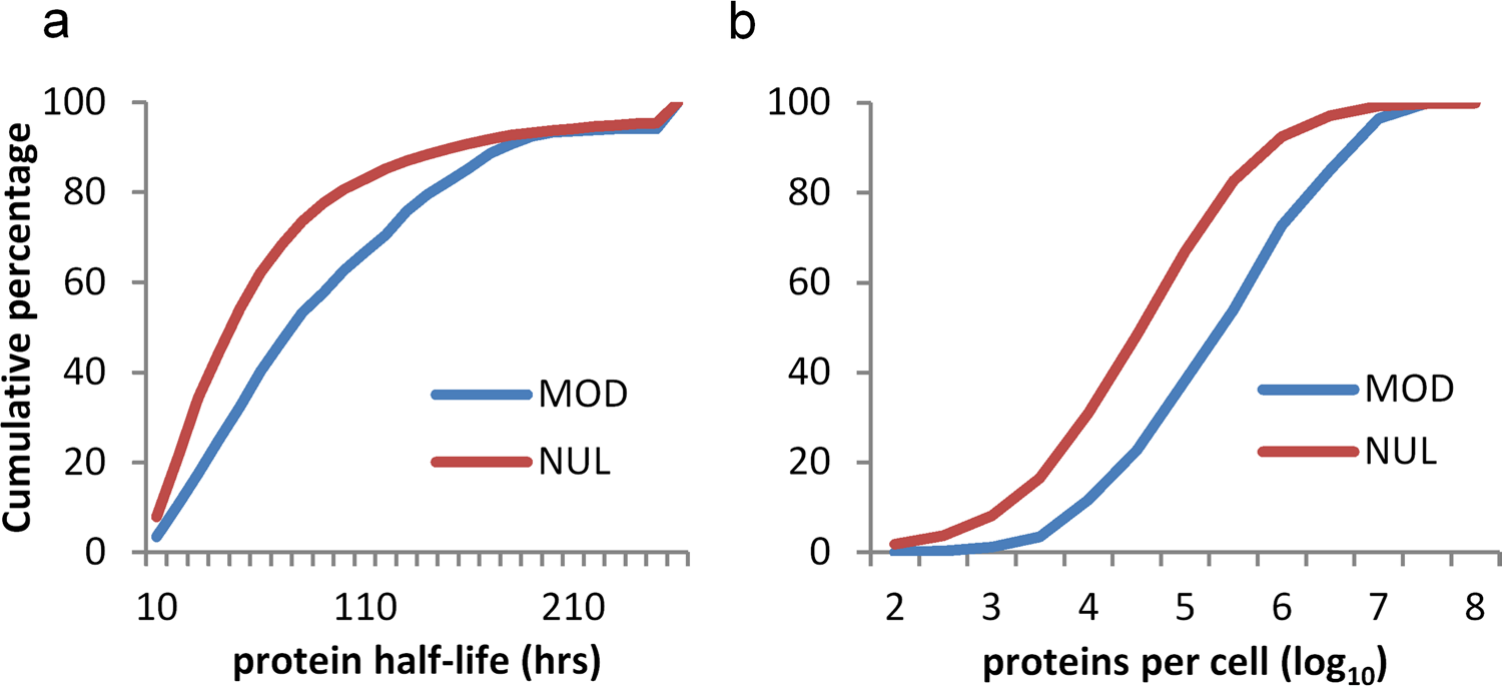
Mouse proteins containing sites of cysteine S-glutathionylation have longer half-lives and greater abundance. (**a**) Cumulative distributions of mouse proteins with measured half-lives (hours)^29^, distinguished as those containing (MOD, 343 instances) or not containing (NUL, 4594) an S-glutathionylated cysteine. (**b**) Cumulative distributions of abundance (log_10_ of protein copies per cell), for proteins with (MOD, 343) or without (NUL, 4609) cysteine S-glutathionylation.

Given that cysteine PTM sites are abundant in regions that are not exposed in 3D-folds, and that they are not enriched in intrinsically disordered regions, it is suggested that the modification of unfolded or partially folded forms may be relatively common. Seeking to establish whether modification is enriched for proteins with lower or higher abundance, or turnover time, glutathione modifications of cysteine were matched with the experimental data that are available for mouse proteins^29^. Mouse proteins with longer half-lives and with higher copy number (molecules per cell) are enriched for cysteine S-glutathionylation (Fig. 6). The inference that unfolded segments of proteins provide a substantial fraction of cysteine PTM substrates, and the observation that protein abundance associates with modification, implies that a degree of non-specificity is evident for cysteine modification, in parallel with the specific redox signalling networks that have been reported^9^.

### Sequences around cysteine PTM sites are enriched for a net positive charge, to a degree that depends on the modification

**Figure 7:**
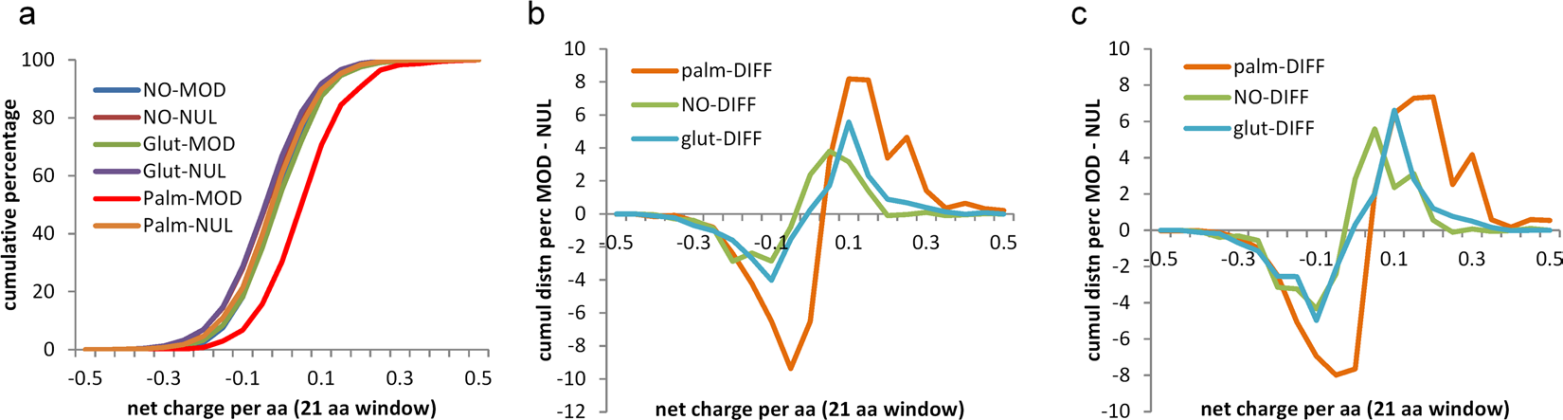
Sequence environment around modified cysteines is enriched in positive charge, especially for S-palmitoylation. (**a**) The net charge per amino acid (in a window of 21 residues centred on cysteine) is shown in averages over modified (MOD) and unmodified (NUL) cysteines, for the three types of modification, with palmitoylated sites showing particular bias towards a positively-charged sequence window. (**b**) Net charge per amino acid is differenced between the averaged data for modified (MOD) and unmodified (NUL) sites, giving MOD – NUL, for each type of cysteine modification. Enrichment for positively-charged environment is seen for S-glutathionylation and S-nitrosylation, although to a lesser degree than for S-palmitoylation. (**c**) The analysis of (**b**) is repeated, but with cysteine modifications from a single species for each PTM: human for S-palmitoylation and S-nitrosylation, mouse for S-glutathionylation.

Sequence analysis allows for study of net charge in a window around modified and unmodified cysteines. The cumulative distributions of net charge per amino acid (with a window of 21 residues) in Fig. 7a show that sites of S-palmitoylation are enriched for surrounding positive charge. Differencing between incremental distributions for modified and unmodified sets, of windowed net charge, highlights the positive charge enrichment around S-palmitoyl sites, and also smaller effects of the same sign for S-glutathionyl and S-nitroso sites (Fig. 7b). The difference plots are replicated in Fig. 7c for data from single species, demonstrating that this observation does not arise from sampling across orthologues.

**Figure 8:**
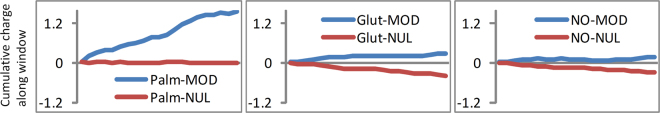
Net charge summed along sequence windows that flank cysteine PTMs. The distribution of net charge along the 21 amino acid window is shown averaged for modified and unmodified sites, for each type of modification.

To test whether enrichments for surrounding net charge are reflected in specific sequence logos, the 21 amino acid windows were aligned at modified cysteines and submitted to the WebLogo tool^30^. No clear single motifs are evident for the modifications with palmitoyl, glutathione, or nitrosyl groups, although an underlying set of sequence motifs (for each modification) could be playing a role in the charge environment^13^. In addition, sequence motifs are known to be important for substrate recognition for certain other cysteine modifications, not studied here (e.g. S-farnesylation^31^). The contribution of each site in the 21 amino acid window around a cysteine site, to net charge, was calculated for both modified and unmodified sites and displayed as a cumulative sum across the window (Fig. 8). A larger positive environment for S-palmitoylation sites (than for S-glutathionylation or S-nitrosylation) is again evident. It is also apparent that positive charge is spread approximately evenly over the window. This cannot simply be a protein net charge effect, since there is a difference to unmodified sites in the same proteins.

Rather than well-populated motifs of a few basic residues, we see an overall basal enrichment for basic over acidic sidechains, over the entire 21 amino acid sequence window around a modification site. We infer that surrounding positive charge, evident from sequence analysis, will tend to shift sites of S-palmitoylation towards the thiolate side of the thiol/thiolate equilibrium, and that this could give increased reactivity in a palmitoyl transfer reaction. It is also possible that enzymatic substrate specificity underlies the positive environment, through a collection of basic motifs distributed around the site of modification.

### Charge environments and chemical mechanisms

Redox potential and pKa values in the thioredoxin superfamily conform to a scheme whereby a more nucleophilic cysteine SG atom is more reactive. Whereas structural data give limited datasets and are inconclusive with regard to pKa predictions, sequence-based results suggest that sites of S-palmitoylation lie, on average, within a positively-charged environment. PATs themselves are acylated by palmitoyl-CoA, and transfer the palmitoyl group between a DHHC domain cysteine and the substrate cysteine^32^. It is possible that increased susceptibility to modification follows substrate nucleophilicity, an effect that could supplement any specificity from cognate PAT/substrate pairings^32^.

Cysteine S-glutathionylation and S-nitosylation sites show much lower enrichment for surrounding positive charge than do sites of S-palmitoylation. Potential mechanisms of S-glutathionylation include direct modification by disulphide exchange with the GSSG/GSH couple, formation *via* other cysteine modifications, and enzymatic (glutathione S-transferase) mediated exchange of glutathione^33,34^. In a direct exchange with the GSH/GSSH couple, the relative nucleophilicity of protein cysteine and GSH thiol group, pKa of 8.7^35^, would play a role. With the GSH thiol pKa above that of a free cysteine sidechain, a positively-charged surround for the substrate cysteine would be less important. Whichever mechanisms for S-glutathionylation are dominant, our results indicate that, in sum, they have a lesser reliance on a positively-charged surround than does S-palmitoylation, and therefore presumably a lower population of substrate cysteine in the thiolate form. Several mechanisms have been reported as leading to protein S-nitrosylation^36^. In terms of charge environment in surrounding sequence, the conclusion is similar to that for S-glutathionylation. If the charge surround is assumed to influence cysteine pKa, then S-nitrosylation mechanisms are generally predicted to act on a lower proportion of thiolate substrate cysteines than do mechanisms for S-palmitolyation.

That a positively-charged surround is relevant for all three modifications, but to varying degrees, is supported by looking at individual modified proteins. For each protein, the question is asked whether the net charge for modified sites, averaged over their 21 amino acid sequence windows, is more positive than the equivalent property for unmodified sites. This calculation yields ratios (in favour of greater positive charge around modified sites), of 4.9:1 (S-palmitoylation), 2.0:1 (S-glutathionylation), and 1.8:1 (S-nitrosylation).

### Regions around cysteine modification are enriched for lysine ubiquitination

**Figure 9:**
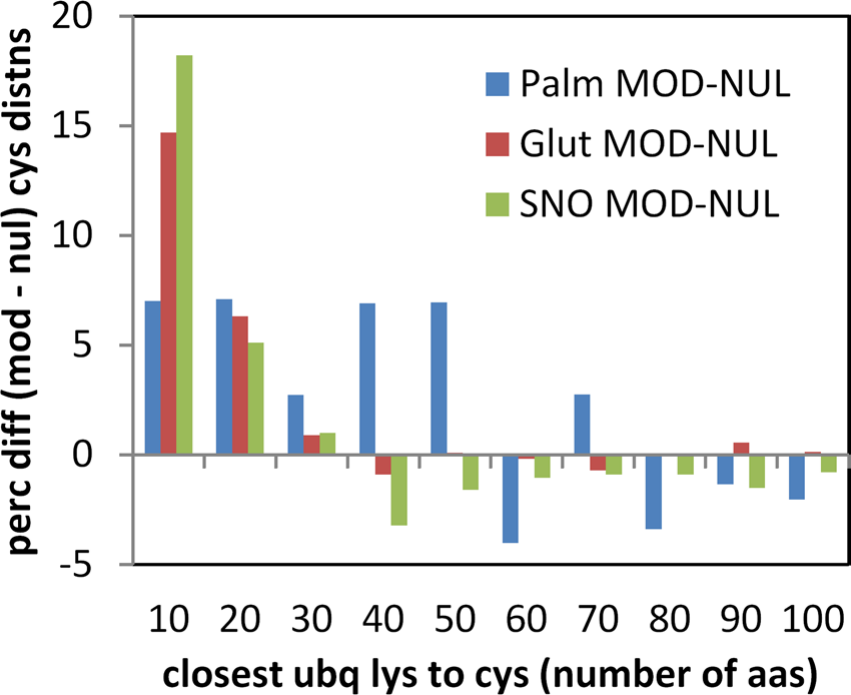
Sequence around modified cysteines is enriched for lysine ubiquitination. For proteins containing both cysteine modification data (for the 3 PTMs), and lysine ubiquitination data^37^, distributions of closest distance between nearest ubiquitinated Lys and Cys were differenced between modified and unmodified cysteine sets. Percentage differences between the distributions (MOD-NUL) are plotted against the Cys to Lys separation in bins of 10 amino acids.

Sequences containing cysteine modifications were cross-referenced with a database of ubiquitination^37^, and distance (number of amino acids) to the closest ubiquitinated lysine recorded for modified and unmodified cysteine. It is apparent that modified sites are, on average, substantially closer to ubiquitinated lysine than are unmodified sites (Fig. 9), in all 3 datasets. The enrichment is most apparent for modified cysteine – ubiquitinated lysine separations within about 30 amino acids, suggesting a relatively localised effect. In order to check whether proximity of cysteine modifications to termini would influence results, the analysis was repeated with exclusion of all cysteines within 50 amino acids of N- and C-termini, which gave similar results.

Structural analysis shows that a surprisingly large fraction of modified cysteines are buried, in at least one structural isoform, for each of the 3 modifications studied. We checked whether modified cysteines known to be solvent inaccessible, were closer to lysine ubiquitination sites, than the equivalent unmofidied cysteines i.e. whether the result of Fig. 9 is maintained for buried cysteine. S-nitrosylation has most data available, with ubiquitination information for 105 of 291 buried and modified cysteines and 128 of 294 accessible and modified cysteines. Percentages were calculated for cysteines within 10 amino acids of a lysine ubiquitination, differenced between modified and unmodified cysteines, (a direct match to a value of 18% in Fig. 9). These values are 20% (accessible cysteines) and 15% (buried cysteines), so that the excess of nitrosylated cysteines close to lysine ubiquitination is maintained for buried sites. Equivalent numbers are too small for meaningful analysis with structurally annotated S-glutathionylations and S-palmitoylations.

The extent to which modified cysteine proximity to ubiquitinated lysine indicates coupling of cysteine modification to the ubiquitin proteasome pathway (UPS) is unknown. It might be expected that natively buried cysteines would be candidates for any such coupling, perhaps flagging partially unfolded regions when modified. However, it is clear that such modification can have physiological relevance for protein biogenesis rather than degradation. For example, C524 in the first nucleotide binding domain (NBD1) of the cystic fibrosis transmembrane conductance regulator (CFTR) is solvent inaccessible (e.g. in 2pze^38^) and S-palmitoylation at this site is important for production of stable protein^39^. CFTR is also known to be ubiquitinated^39^, but the closest modified lysine in the available data is 164 amino acids distant, with no published report of coupling between S-palmitoylation and ubiquitination. CFTR NBD1 has been shown to undergo a vectorial folding process^40^, which could account for exposure of C524 during protein biogenesis.

The CFTR S-palmitolyation example shows that modification of natively buried cysteines, in proteins that also known to be ubiquitinated, could couple with protein biogenesis and function rather than protein degradation. S-palmitoylation is likely to be a different case generally to S-glutathionylation and S-nitrosylation, due to its role in membrane targeting of proteins. The signal for modified cysteine proximity to ubiquitinated lysine is larger for S-glutathionylation and S-nitrosylation, than for S-palmitoylation (Fig. 9), and here there is some evidence for a link with protein degradation, as is the case for phosphorylation^41^. S-nitrosylation of phosphatase and tensin homolog (PTEN) promotes its ubiquitin-dependent degradation by the proteasome^42^, as is also the case for phosphodiesterase type 5 (PDE5)^43^. A role for redox reactions and high levels of NO leading to ubiquitination and proteasomal degradation has been discussed^44^, as has proteosomal-mediated quality control of S-nitrosylated mitochondrial proteins^45^. Complicating factors in experimental analysis of any coupling between cysteine oxidations and protein degradation include their reversibility^46^, and the absence of a consensus ubiquitination sequence motif^47^. The data shown in Fig. 9, allied to the observation that modification of natively buried cysteines is common, are consistent with an association of cysteine modification and lysine ubiquitination in some cases. Further proteomics investigation is required to establish whether such association is simply based on a common substrate property (such as protein disorder), or whether it plays a role in targeting, perhaps towards UPS-mediated protein degradation.

## Discussion

**Figure 10:**
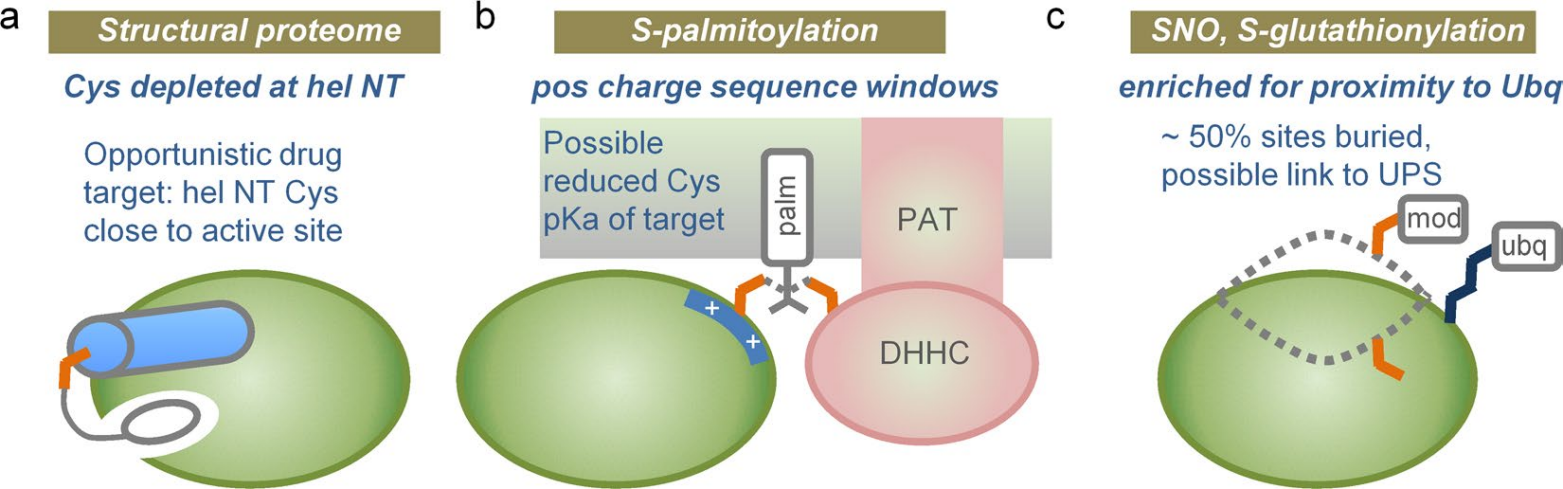
Bioinformatics insights to cysteine reactivity. (**a**) Depletion of cysteine at α-helical amino-termini, relative to aspartic acid and serine, suggests that cysteine reactivity is modulated in evolution. Where Cys at a helical N-terminus does occur, and where this is adjacent to an enzyme active (or other modulatory) site, scope exists for targeting with covalent inhibitors. (**b**) S-palmitoylation sites in particular are enriched for a positively-charged sequence surround, which could lower the Cys pKa, leading to more favourable transfer from the DHHC domain of a PAT. (**c**) Most notably for sites of S-nitrosylation and S-glutathionylation, about half of modified sites are buried in native structure, and sites are enriched for proximity to lysine ubiquitinations. One possibility is that cysteine modification could be linked to UPS-mediated degradation.

A schematic summary of our results is given in Fig. 10. We commenced with the physicochemical considerations that lead to the well-characterised cysteine location at α-helix amino termini in the thioredoxin family. Indeed, a relative depletion of cysteine at this site in a representative set of 1627 human protein structures suggests that sequences may have evolved to depress background cysteine reactivity (Fig. 10a). This biophysical view is enhanced with prediction of enrichment for lower pKas (and increased nucleophile-based reactivity) for cysteines at α-helix amino termini in the hu1627 set. Further, an amino-terminal cysteine is a key site of covalent linkage for a pharmaceutically-relevant class of kinase inhibitors (Fig. 10a). Generally, pKa predictions could aid in the search for other reactive cysteines, adjacent to active sites, interfaces, or potential allosteric effector sites.

The study progressed to large-scale datasets of three cysteine modifications, S-palmitoylation, S-glutathionylation, and S-nitrosylation. In common with other work^9^, it is found that the biophysical basis of reactivity is not clear-cut, in contrast to the thioredoxin family. There may be some enrichment for modification at sites with lower predicted cysteine pKas, but the attrition though structural annotation leads to small numbers on which to base this assessment. A striking finding is that modified sites are distributed in almost the same fractions between solvent accessible and inaccessible, as are unmodified sites in the same proteins. The criterion for solvent inaccessibility was strict (zero SASA). Looking at sequences rather than structures, two observations are clear: intrinsically disordered regions are not enriched around modified cysteines (unlike phosphorylation sites), and modified sites are located in sequence regions of net positive charge, particularly the case for S-palmitoylation. It was also found that, for mouse protein S-glutathionylation, modified proteins tend to have longer half-lives and higher abundance in the cell. With regard to the charge environment, one interpretation is that physicochemical reactivity could indeed play a role for S-palmitoylation, with reduced cysteine pKa and increased nucleophilicity contributing to modification (Fig. 10b). There may be a smaller effect along these lines for S-glutathionylation and S-nitrosylation. It is unclear how altered cysteine reactivity intersects with substrate specificity for S-palmitoylation mediated by DHHC S-acyltransferases^32^, or with wider redox signalling networks based on cysteine modification^9^.

Finding that a significant fraction of modified cysteines are buried in their native structure suggests that they are in unfolded, mis-folded, or partly folded regions when modified. In principle, this could arise from a scrambling of modification sites in the analytical procedure, rather than from a physiological population. However, there are no reports of this occurring on the scale required to explain the fraction of buried sites seen in the 3D part of our analysis, although the problem has been discussed in the context of quantitative proteomics^48^. In addition, in the datasets used here, there are large excesses for certain gene ontology (GO) classifications, particular those associated with membrane. Giving percentage of modified subset *versus* percentage of organism population (human or mouse) annotated with a particular GO term^49^: S-palmitoylation, plasma membrane, 76.3%,13.8%; S-glutathionylation, membrane-bounded organelle 84.9%, 46.1%; S-nitrosylation, membrane-bounded organelle 84.9%, 36.3%. It is interesting that a large fraction of modified proteins are associated with membranes. This might be expected from the functional viewpoint, whether at the plasma membrane for S-palmitoylated proteins, or in organelles for S-glutathionylation and S-nitrosylation, where redox-based metabolism will be particularly important. Future work could benefit from more extensive calculation^50^ and analysis^51^ of features associated with peptide motifs around sites of post-translational modification. It would also be advantageous to enhance analysis of redundancy between proteins containing motifs^52^, and integrate PTM targets into protein-protein interaction networks^53^.

Lysine ubiquitination targets proteins for proteasomal degradation, with some indication of a substrate preference for disordered regions^54,55^. For the modifications studied here, cysteine PTMs appear to be linked as much with unfolded as with folded regions. Enrichment for proximity to ubiquitinated lysine suggests that a subset of cysteine PTMs is associated in some way with protein targeting *via* ubiquitination (Fig. 10c). It is possible that combining data on cysteine (and other amino acid) modifications, with lysine ubiquitination, could reveal regions of protein that are natively inaccessible, but become exposed and potentially aggregation-prone. Such information could prove valuable in understanding protein quality control and in biotechnological production of proteins. Post-translational modifications at sites other than cysteine are known to assist recognition of protein degrons^56^. Whether the proximity of cysteine and lysine modifications simply reflects a common substrate (e.g. an unfolded protein region), or whether it arises from an ordered process of one modification followed by another, perhaps in a variation of targeting for degradation, remains to be established.

## Methods

### A representative set of human protein structures

From a listing of source species for each protein structure in the protein databank (PDB, www.rcsb.org ref.^57^,) of early 2016, the term ‘HOMO SAPIENS’ returned 30,631 entries. In order to reduce the redundancy of structures, the list was cross-referenced with reduction at the 25% sequence identity level, using the PISCES online tool for protein chains in each structure file^58^, additionally requiring better than 2.5 Å resolution and 1.0 R-factor, yielding 1672 structures. Computational requirements of the pKa predictions increase substantially at larger protein size and number of ionisable groups, and a filter for asymmetric units with less than 1500 amino acids was applied, excluding only 45 of the structures, leaving 1627. This dataset (termed hu1627) was used to study the range of predicted pKas for cysteines in human proteins, together with environmental information for cysteine sites. Equivalent cysteine pKas were averaged in an asymmetric unit, and code was written to interrogate PDB files for location of amino acids in annotated helices and β-strands, as well as the presence of heteroatoms within 4 Å of the cysteine sidechain SG atom. Secondary structure annotation in the structure files was used to compute relative propensity for an amino acid at a given secondary structure location. This is calculated as relative amino acid occupancy,

$${{\rm{R}}}_{\mathrm{aa},\mathrm{loc}}=[({{\rm{P}}}_{{\rm{aa}},{\rm{loc}}}/{{\rm{P}}}_{{\rm{aa}},{\rm{all}}})-1],$$

where P_aa,loc_ is the percentage composition of an amino acid at a given location (e.g. first in an α-helix), and P_aa,all_ is the percentage composition for the same amino acid, summed over all locations. Thus, R_aa,loc_ gives a signed indication of enrichment (positive) or depletion (negative) of an amino acid at a given location. Of particular interest were these values for locations within an α-helix, for cysteine and comparator amino acids (serine, aspartate and glutamate). Amino acids were assigned to either the N-terminal or C-terminal region of a helix, not to both, with the transition occurring at the midpoint of the helix.

### Human protein kinase structures

To obtain a representative set of human protein kinase structures, 509 human protein kinase^59^ IDs were obtained from UniProt (http://www.uniprot.org/docs/pkinfam ^60^). These IDs were matched to the results from a search of the PDB for X-ray crystal structures of human protein kinases, with a non-redundancy filter set at 100% amino acid identity, so that just one copy of structures with identical sequence and length was processed. A single chain, mapped to a UniProt protein kinase ID, was extracted from each PDB file for calculation. Further focus was applied to EGFR kinases, with a subset of 143 extracted with a text search for EGFR from the PDB, without any sequence redundancy filter, allowing study of structural variation around the C797 site of modification^20^.

### Structure-based pKa prediction methods

Predictions of pKas were made with the FDDH method, previously assessed to have a root mean square deviation to experimental values in a benchmark set of 0.8 pKa units^61^. Relative dielectric values of 78.4 for solvent and 4 for protein were used, with an ionic strength of 0.15 M, and model compound pKas of Asp 4.0, Glu 4.4, Lys 10.4, Arg 12.0, His 6.3, Cys 8.3, N-terminus 7.5 and C-terminus 3.8. Tyrosine has a model compound pKa of 10.2, so that it generally acts as a buried or surface polar group rather than a charged group, and was included only in the sidechain hydroxyl form. The pKa prediction pipeline includes a torsional adjustment of OH groups, such that optimal interaction is made with the ionised states of Asp, Glu, Lys, Arg, His and Cys sidechains^61^. Interactions between charge components were used in a statistical mechanical scheme describing protonation energetics^62^, with Monte Carlo sampling of protonation states^63^ to derive predicted pKas. Code was written to link pKa predictions to structural information that was derived from PDB files. Information directly available includes secondary structure location from HELIX and SHEET records and disulphide bond status from the SSBOND record. Calculations were made to obtain the number of heteroatoms within 4 Å of a cysteine SG atom, and SASA, using in-house software. An additional disulphide status check was made, using a screen for pairs of SG atoms within 2.8 Å of each other. For the pKa predictions, all cysteines were treated as if free from disulphide bonds, to permit ionisation. Data were stored with property flags, such that either the complete dataset or subsets with selected characteristics (e.g. cysteine at the amino-terminus of an α-helix) could be extracted. Multiple sequence alignment for selected protein kinases was made with an implementation of Clustal^64^ at the European Bioinformatics Institute^65^. Structures were visualised with PyMOL (www.pymol.org) and Swiss-PdbViewer^66^.

### Datasets for post-translationally modified cysteines

Three cysteine PTMs were the focus for this study. Several resources have collated PTMs, including modified cysteines (e.g. dbPTM^67^). UniProt is a convenient resource for cross-referencing protein identifiers (including protein structure), and for retrieving data, including PTM information^60^. Criteria used in retrieval were to obtain data (covering multiple species) that could be easily cross-referenced with UniProt identifiers, and that when representatives for a single species were extracted, there remained a sizeable fraction of the dataset. This latter criterion allows results to be checked when orthologues are excluded, so that any potential annotation by similarity, between species, is removed. A text file copy of the January 2016 UniProt database of protein sequences and annotations was interrogated for cysteine modifications. For the three PTMs studied, 3867 instances of S-palmitoyl cysteine were returned, 540 of S-nitrosocysteine, and 66 of S-glutathionyl cysteine. Since the number of S-glutathionylation instances is relatively small, further data were sought and added from the redoxDB database (243 instances^68^), and from proteomic studies (890 instances^9^, and 353 instances^69^), for a total of 1552. Filtering UniProt S-nitrosocysteine modifications for human leaves just 60 proteins, so that an alternative was sought. From the October 2016 dbSNO^70^, 4165 cysteine nitrosylation sites were retrieved.

Removing redundancy and requiring that windows of 21 amino acid length could be centred at each site, and which therefore excluded protein termini in the sequence-based calculations, reduced the numbers to 2598 S-palmitoylated (Supplementary Table S1), 1166 S-glutathionylated (Supplementary Table S2), and 3547 S-nitrosylated (Supplementary Table S3) sites. Numbers of cysteines, in these sets of proteins, that are not reported to be modified, and are approximated to be negative examples for comparative analysis are, 26330 (S-palmitoylation, Supplementary Table S4), 8900 (S-glutathionylation, Supplementary Table S5), and 15517 (S-nitrosylation, Supplementary Table S6). The orthologue-free subsets (again excluding protein termini) consisted of 320 S-palmitoylations (human), 968 S-glutathionylations (mouse), and 953 S-nitrosylations (human).

Sequences and sites of sequence modification from the multiple species sets of the three cysteine modifications were associated with protein structures, where possible, using in-house code developed to use the SIFTS resource for mapping UniProt sequence numbers to PDB residue numbers^71^. Structural coverage of specific regions in proteins (excluding the use of comparative models) is still rather limited, and this process yielded the following numbers of positive and negative modification sites: 23, 234 S-palmitoylation, 114, 401 S-glutathionylation, and 585, 1654 S-nitrosylation, for which FDDH pKa calculations were made. Calculations were made on asymmetric unit PDB files, and results averaged between equivalent sites in multiple chains. Since some of the structurally annotated datasets are small, sequence analysis was also developed.

### Sequence-based calculations

Sequences were analysed with 21 and 11 amino acid windows centred on modified and unmodified cysteine sites, summing the net charge around each site. With physiological pH around 7, and considering model compound pKas, sidechain charges were assumed as −1 for aspartic and glutamic acids, and +1 for lysine and arginine, with histidine neutral. This is a lower resolution approach than the structure-based analysis, but it leverages the large number of cysteine modification sites evident from mass spectrometry, whereas the structural annotation process has a high rate of attrition. The approximation that unmodified sites in any protein that contains a modified (positive) site, are negative examples^72^, is unlikely to be accurate in all cases, but the aim is to detect enrichments of environment for modification in a population of sites. Motifs around modified cysteines were constructed by supplying 21 amino acid windows, aligned at the cysteine modification site, to the WebLogo tool (http://www.weblogo.berkeley.edu)^30^. In addition, code was written to calculate the net charge at neutral pH at each location within a window, averaged over sequences in each subset of modified or unmodified sites. Further calculations were made to rank the net charge in 21 amino acid windows for each protein, so that modified sites could be compared with unmodified sites in a protein. Thus, for each protein, the extremes of net charge in 21 amino acid windows (modified and unmodified sites) were found, and then each site positioned within this range. The average location (net charge) for modified sites could then be compared with that for unmodified sites, within a single protein.

Predictions of folding propensity were made using the separation of folded and intrinsically unfolded states developed from a combination of hydropathy and net charge^27^, and incorporated into in-house code. This propensity was calculated over the 21 amino acid windows around cysteine (modified and unmodified) sites. In order to assess these windowed calculations against benchmark values, datasets of known ordered and disordered proteins were retrieved^28^. Data for mouse protein half-lives and copy numbers (molecules per cell) were obtained from a global study of gene expression control^29^.

A dataset of lysine ubiquitinations in mammals (data_2013_10_12.rar, mUbiSiDa^37^) was retrieved. Code was written to cross-reference cysteine modifications with lysine ubiquitination in the same protein, and derive cys-lys separations. Searches for gene ontology terms enriched within subsets of proteins were made with the Princeton GO-TermFinder software (http://go.princeton.edu/cgi-bin/GOTermFinder)^49^.

### Code availability

Custom code developed for this study is available by request from the authors.

## Electronic supplementary material

Supplementary Information
